# Pharmacological Characterization of a Recombinant Mitochondrial ROMK2 Potassium Channel Expressed in Bacteria and Reconstituted in Planar Lipid Bilayers

**DOI:** 10.3390/membranes13030360

**Published:** 2023-03-21

**Authors:** Milena Krajewska, Adam Szewczyk, Bogusz Kulawiak, Piotr Koprowski

**Affiliations:** 1 Laboratory of Intracellular Ion Channels, Nencki Institute of Experimental Biology PAS, 02-093 Warsaw, Poland; 2 Interdisciplinary Laboratory of Molecular Biology and Biophysics, Centre of New Technologies, University of Warsaw, 02-097 Warsaw, Poland

**Keywords:** ROMK2, mitoK_ATP_ channel, polymer nanodiscs, planar lipid bilayer, potassium channel modulators

## Abstract

In the inner mitochondrial membrane, several potassium channels that play a role in cell life and death have been identified. One of these channels is the ATP-regulated potassium channel (mitoK_ATP_). The ROMK2 potassium channel is a potential molecular component of the mitoK_ATP_ channel. The current study aimed to investigate the pharmacological modulation of the activity of the ROMK2 potassium channel expressed in *Escherichia coli* bacteria. ROMK2 was solubilized in polymer nanodiscs and incorporated in planar lipid bilayers. The impact of known mitoK_ATP_ channel modulators on the activity of the ROMK2 was characterized. We found that the ROMK2 channel was activated by the mitoK_ATP_ channel opener diazoxide and blocked by mitoK_ATP_ inhibitors such as ATP/Mg^2+^, 5-hydroxydecanoic acid, and antidiabetic sulfonylurea glibenclamide. These results indicate that the ROMK2 potassium protein may be a pore-forming subunit of mitoK_ATP_ and that the impact of channel modulators is not related to the presence of accessory proteins.

## 1. Introduction

The ATP-regulated potassium (mitoK_ATP_) channel, first identified in the inner mitochondrial membrane of rat hepatocytes cells [1], has since been found in various other tissues, including the heart [2,3], brain [4,5,6], kidney [7], skeletal muscles [8], human T lymphocytes [9], and also skin fibroblasts [10]. Electrophysiological techniques such as patch-clamp of mitoplast membranes and reconstitution of inner mitochondrial membranes into a planar lipid bilayer have been utilized for the pharmacological characterization of the mitoK_ATP_ channel [1,11,12,13,14]. Despite these efforts, the molecular identity of the mitoK_ATP_ channel remains a topic of ongoing debate. The initial hypothesis proposed that the Kir6.x channel complex played a role in mitoK_ATP_ activity. However, subsequent studies indicated that the deletion of the genes encoding Kir6.1 and Kir6.2 did not eliminate mitoK_ATP_ activity [15,16]. Alternative theories suggest that the mitoK_ATP_ channel may involve a succinate dehydrogenase complex [17]. Currently, there are three hypotheses regarding the protein identity responsible for functioning as the mitochondrial K_ATP_ channel.

In the first hypothesis, Foster et al. [18] proposed that the ROMK2 protein, encoded by the KCNJ1 gene, serves as the molecular component of the mitoK_ATP_ channel in rat heart mitochondria. They demonstrated that the first 24 amino acids of ROMK2 contain a mitochondrial targeting sequence sufficient to import green fluorescent protein into mitochondria and that the overexpression of ROMK2 in H9c2 cells confers protection against oxidative stress-induced cell death [18]. Subsequent patch-clamp studies have associated overexpression of ROMK2 with mitoK_ATP_ activity in H9c2 cells. These channels were found to be inhibited by 5-hydroxydecanoic acid (5-HD) and ATP/Mg^2+^ (mitoK_ATP_ inhibitors) as well as by Tertiapin Q (ROMK inhibitor). Conversely, diazoxide (mitoK_ATP_ channel opener) increased the open probability of recorded channels [11].

The second hypothesis presented in studies in 2019 suggests that the product of the CCDC51 gene dubbed mitoK serves as the pore-forming subunit of the mitoK_ATP_ [14]. Additionally, it was proposed that ABCB8 serves as a regulatory subunit of the mitoK_ATP_ channel, making it sensitive to the drug glibenclamide. As such, this protein was referred to as the mitochondrial sulfonylurea receptor (mitoSUR).

The third hypothesis, which is more recent, proposes that the ATP-synthase enzyme, present in the inner mitochondrial membrane, plays a role in mediating not only H^+^ but also K^+^ fluxes in order to drive ATP synthesis [19,20].

The action of modulators on mitochondrial potassium channels is a subject of ongoing controversy and is often indirect in nature. This is particularly evident when studying these channels in intact cells or isolated functional mitochondria. The wide range of side or non-specific effects associated with potassium channel modulators further complicates the interpretation of mitochondrial potassium channels’ role in various tissues. To gain a better understanding of the pharmacological properties of mitochondrial potassium channels, it is crucial to study them in a well-defined and controlled protein and lipid environment.

In previous studies, we have successfully employed a heterologous expression system in *Escherichia coli* and polymer nanodiscs to obtain partially purified ROMK2-6xHis potassium channels, which were subsequently characterized in planar lipid bilayers (PLBs) [21,22]. In the present study, we used this same system to investigate the impact of mitoK_ATP_ channel modulators on the activity of ROMK2 potassium channels in absence of contamination by any eukaryotic proteins. Our findings indicate that the pharmacological profile of observed activity in the planar lipid bilayer is partially consistent with that of the mitoK_ATP_ channel. Interestingly, we found that glibenclamide affects the function of the ROMK2 channel in the absence of mitoSUR.

## 2. Materials and Methods

### 2.1. Chemicals

Styrene-maleic acid copolymer SMA 2.3:1 (brand name XIRAN^®^; M_n_ 3000 g mol^−1^; M_w_ 6500 g mol^−1^) was a gift from Polyscope (Geleen, The Netherlands). Azolectin (L-α-phosphatidylcholine from soybeans, Type II-S), adenosine 5′-triphosphate magnesium salt (ATP/Mg^2+^), glibenclamide, diazoxide, and 5-hydroxydecanoic acid (5-HD) were purchased from Sigma-Aldrich. VU591 was purchased from Alomone Labs (Jerusalem, Israel).

### 2.2. Preparation of Polymer Nanodiscs Containing ROMK2 Protein

**Figure 1:**
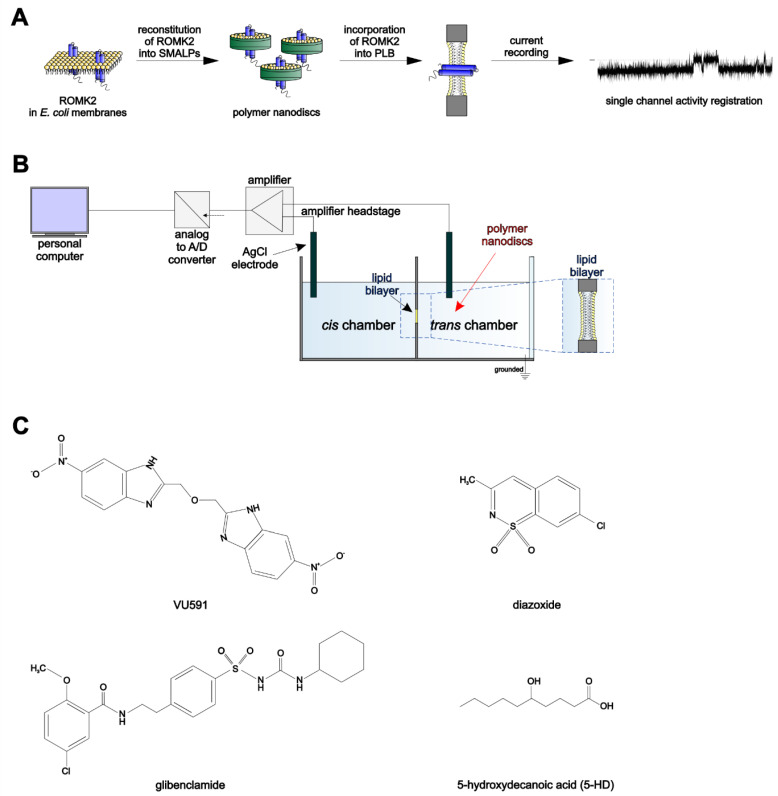
(**A**) Schematic representation of the experiment. From left to right: (i) Reconstitution of the ROMK2 channel protein from *E. coli* membranes into polymer nanodiscs (SMALPs), (ii) Incorporation of ROMK2 from the protein-containing nanodiscs into a planar lipid bilayer, (iii) Recording of single channel activity. (**B**) Diagram of the complete bilayer setup. Experimental data were collected and processed based on the current recorded between two compartments—*cis* and *trans*. The *trans* compartment was composed of a Teflon cup holder with a 250 μm aperture. Both compartments were connected by agar salt bridges and Ag/AgCl electrodes with an amplifier, an analog-to-digital converter, and a personal computer (PC). The compartments were filled with symmetrical (50/50 mM KCl) or asymmetrical 50/150 mM KCl solutions (cis/trans, respectively). SMALPs containing ROMK2 protein were added to the trans compartment. (**C**) Chemical structures of modulators of mitoK_ATP_ or ROMK2 channels.

The preparation of polymer nanodiscs containing the ROMK2 protein was carried out as previously described [21]. Briefly, *E. coli* membranes containing heterologously expressed ROMK2 protein (final concentration 45 mg/mL) were solubilized with 2.5% SMA 2.3:1 copolymer in a buffer containing 50 mM Tris/HCl, 200 mM KCl, 1 mM EDTA pH 7.4 supplemented with protease inhibitor cocktail (cOmplete™, Roche, Mannheim, Gemany), and 1 mM PMSF. The suspension was then incubated overnight at 25 °C with gentle shaking. The following day, the suspension was centrifuged at 100,000× *g* for 1 h at 4 °C to remove the insoluble fraction. The supernatant was then subjected to metal affinity chromatography using His-Select^®^ Nickel Affinity Gel (Sigma-Aldrich, St. Louis, MO, USA, cat. P6611). The ROMK2-containing nanodiscs were eluted with 250 mM imidazole and subsequently dialyzed using a 3.5 kDa NMWCO dialysis membrane against the buffer containing 300 mM KCl and 50 mM Tris/HCl, pH 7.4 to remove imidazole. The resulting nanodiscs were aliquoted and stored at −80 °C. These polymer nanodiscs containing ROMK2 protein were subsequently used for planar lipid bilayer reconstitutions and activity measurements, as illustrated in Figure 1A.

### 2.3. Planar Lipid Bilayer (PLB) Measurements

Planar lipid bilayer (PLB) measurements were conducted as described previously [6,21,23,24]. The experiments were performed at room temperature. The electrical connections were made by agar salt bridges (3 M KCl) and silver/silver chloride electrodes (Ag/AgCl). The *trans* compartment was grounded, and the voltage was applied to the *cis* compartment. To minimize the influence of electromagnetic disturbances, the entire measurement system was placed in a Faraday cage. In addition, the entire measuring system was placed on an anti-vibration table to protect it against the effects of mechanical vibrations. Lipid bilayers were formed with azolectin in n-decane (25 mg/mL) in a ~250 μm aperture in the wall of a Teflon cup (Warner Instrument Corp., Hamden, CT, USA) separating two chambers: *cis* and *trans* (1 mL of internal volume each). The formation and thinning of the lipid bilayers were monitored by capacitance measurement and deemed adequate for leak conductance of less than 2–3 pS when assessed with a −70 and 70 mV voltage. The typical capacitance of the bilayer was 90–150 pF. The *cis* and *trans* compartments contained 150 mM KCl solutions in symmetrical or respectively 50 and 150 mM KCl in asymmetrical conditions buffered with 10 mM 4-(2-hydroxyethyl)-1-piperazineethanesulfonic acid (HEPES), pH 7.4. Native nanodiscs containing ROMK2 protein were added to the *trans* compartment and gently stirred until the channel incorporation was observed (Figure 1B). Incorporation of the ROMK2 protein into the PLB occurred within a few seconds to several minutes. The studied compounds (Figure 1C) were added to both or successively to *cis* and *trans* compartments. The current was measured using a Bilayer Membrane Amplifier (BLM-120, BioLogic, Orlando, FL, USA)

### 2.4. Data Analysis

The current was filtered at a frequency of 100 Hz and digitized at a sampling rate of 500 kHz using an A/D converter Power Lab 2/25 (ADInstruments, Sydney, Australia). The data was then transferred to a PC for analysis by Chart v5.2 (PowerLab AdInstruments, Sydney, Australia). The Clampfit10.7 software (Molecular Devices, San Jose, CA, USA) was used for data processing. Single-channel currents were recorded at various voltages, and the channel conductance was calculated based on the current-voltage relationship. In symmetrical systems, the current-voltage relationship was approximately rectilinear fulfilling Ohm’s law. In unsymmetrical conditions, a rectilinear range with the negative values of the applied voltage was used to determine the conductance of the channel. The effect of substances on ROMK2 channel activity was tested at a voltage of −70 mV. The channel open probability, nP(o), was calculated using the Single-Channel Search module with the Clampfit 10.7 program, which allows for determining the closed and open levels. The illustrated channel recordings are representative traces of the given conditions. Data are presented as the mean ± standard deviation (SD). Statistical analysis was performed with Student’s *t*-test (pair sample *t*-test, Origin) or one-way ANOVA with Tukey’s test (posthoc test), and *p*-values below 0.05 were considered significant (* *p* < 0.05; ** *p* < 0.01; *** *p* < 0.001; **** *p* < 0.0001).

## 3. Results

### 3.1. Identification of ROMK2 Single-Channel Activity

**Figure 2:**
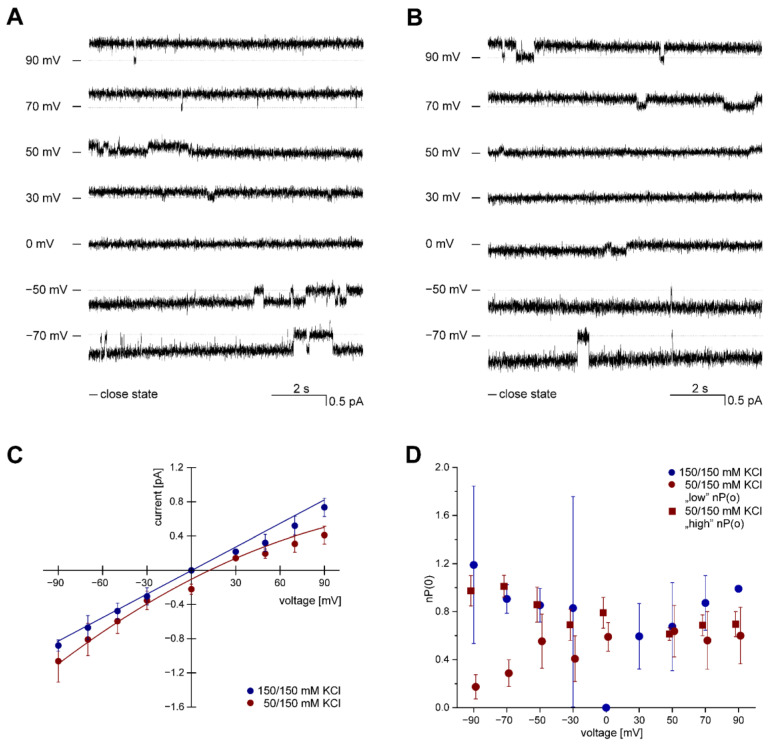
Functional characterization of the activity of ROMK2 channel reconstituted from polymer nanodiscs into planar lipid bilayers. (**A**) The representative activity of the ROMK2 channel at different voltages in symmetrical conditions. (**B**) The representative activity of the ROMK2 channel at different voltages in asymmetrical conditions. (**C**) Current-voltage characteristics of single ROMK2 channels in symmetrical (navy blue) (*n* = 5) and asymmetrical (burgundy color) (*n* = 45) conditions. (**D**) The dependence of the open probability of ROMK2 channels on voltage in symmetrical (navy blue) and asymmetrical (burgundy color) conditions. The channels that were recorded under asymmetrical conditions, based on their activity at negative voltages, were classified into two distinct groups: one with low nP(o) (represented by circles, *n* = 9) and the other with high nP(o) (represented by squares, *n* = 13). “-” indicates a closed state of the channel.

This work is an extension of our previous study and uses the same preparation [21] of native nanodiscs (SMALPs) containing the ROMK2 protein for the incorporation of active channels in planar lipid bilayer (PLB) membranes (as illustrated in Figure 1A). Following nanodisc fusion with the lipid bilayer, the current characteristic of ion channel activity was observed. Figure 2A presents single-channel recordings in symmetric (150/150 mM KCl *cis*/*trans*) solution at various voltages, while Figure 2B illustrates representative single-channel recordings of the channel active in asymmetric (50/150 mM KCl *cis*/*trans*) conditions. Analysis of the current-voltage relationship revealed the calculated conductance of 11 pS ± 0.44 pS (*n* = 5) for the ROMK2 channel in the symmetrical conditions and 9.0 pS ± 0.92 pS (*n* = 48) under gradient conditions (50 mM/150 mM KCl). The mean reversal potential under these conditions, as determined by fitting the curve to the experimental data at negative voltage values, was 16 mV ± 5.3 mV (Figure 2C). In asymmetric conditions, the rectification of the current was observed. The mean open probability of the channel, nP(o), remained similar throughout the tested voltage range (Figure 2D).

### 3.2. Pharmacological Modulation of the ROMK2 Channel Activity

To investigate the ion channel properties observed in our experiments, we utilized known modulators of ROMK and mitoK_ATP_ channels at their previously reported effective concentrations. Because ROMK2 channel conductance is relatively small, we opted to study the impact of modulators on channel activity in asymmetric conditions at −70 mV, at which channel openings are readily visible. We observed two populations of ROMK activities characterized by high or low open probabilities under these conditions (Figure 2D). At extreme open probabilities, the effects of modulators might not be observed, i.e., when activators are tested on highly active channels and vice versa. Therefore, we chose channels with low open probability to test impact activators and channels characterized by high probability to test the influence of inhibitors.

**Figure 3:**
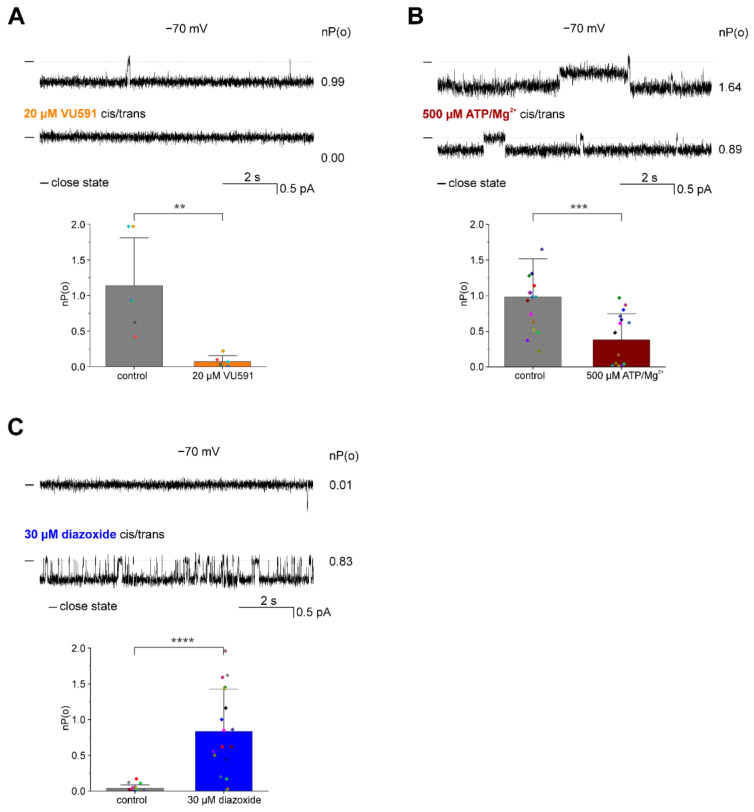
Pharmacological characterization of the activity of ROMK2 channel protein reconstituted in planar lipid bilayers. (**A**) VU591, a known inhibitor of the ROMK channel, inhibits recorded channel activities. Top panel: representative single-channel recordings at −70 mV under control conditions and in the presence of 20 µM VU591. Lower panel: channel open probability, nP(o), under control conditions and in the presence of 20 μM VU591 (*n* = 6). (**B**) ATP/Mg^2+^, a known mitoK_ATP_ and ROMK channel inhibitor, blocks recorded channel activities. Top panel: Representative single-channel recordings at −70 mV under the control conditions and in the presence of 500 μM ATP/Mg^2+^. Lower panel: channel open probability, nP(o), under the control conditions and in the presence of 500 μΜ ATP/Mg^2+^ (*n* = 16). (**C**) Diazoxide, a known mitoK_ATP_ channel opener, increases observed channel activity. Top panel: Representative single-channel recordings of ROMK2 at −70 mV under the control conditions and after the addition of 30 µM diazoxide. Lower panel: channel open probability, nP(o), under the control conditions and in the presence of 30 μM diazoxide (*n* = 18). “-” indicates the closed state of the channel. The results related to channel open probability are presented as means ± SD. ** *p* < 0.01, *** *p* < 0.001, **** *p* < 0.0001.

Firstly, PLB experiments were performed to test the impact of VU591, a specific synthetic blocker of ROMK channel activity [21,25,26]. Previous studies have shown that VU591 completely inhibits ROMK channels at 10 µM and has no significant impact on other Kir channels at concentrations up to 50 µM. As shown in Figure 3A, the addition of 20 µM VU591 resulted in a virtually complete block with a decrease in the open probability of the recorded channels from 1.14 ± 0.67 to 0.07 ± 0.08 (*n* = 6).

We also observed a significant inhibition of ROMK activity by ATP, a specific blocker of mitoK_ATP_ channels [10,27] (Figure 3B). The addition of 500 µM ATP/Mg^2+^ applied to both sides of the lipid bilayer resulted in a decrease in the channel open probability from 0.98 ± 0.53 to 0.38 ± 0.36 (*n* = 16).

We also tested diazoxide, a known activator of mitoK_ATP_ [28]. Previous flux assays [18] and patch-clamp recordings from mitochondria [11] suggested that diazoxide (30 µM) also activates the ROMK2 channel. However, since ROMK channels could interact with regulatory subunits, those experiments did not provide evidence of a direct effect of diazoxide on the activity of the pore subunit of this channel. In this study, we show that the addition of 30 µM diazoxide from both sides of the lipid bilayer resulted in a significant increase in the open probability of ROMK2 channels from 0.04 ± 0.05 to 0.84 ± 0.59 (*n* = 18) (Figure 3C).

**Figure 4:**
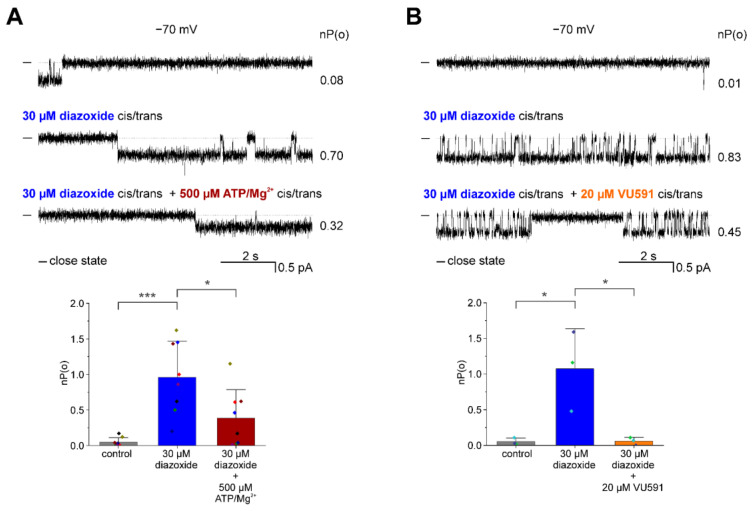
Inhibition of diazoxide-activated ROMK2 channels. (**A**) Inhibition of the diazoxide-activated channel by 500 µM ATP/Mg^2+^. Top panel: Representative single-channel recordings at −70 mV under control conditions, in the presence of 30 µM diazoxide, or 30 µM diazoxide and 500 µM ATP/Mg^2+^. Lower panel: channel open probability nP(o) under the control conditions, after the addition of 30 µM diazoxide, or 30 µM diazoxide and 500 µM ATP/Mg^2+^ (*n* = 8). (**B**) Inhibition of the diazoxide-activated channel by VU591. Top panel: Activation of the ROMK2 channel by 30 µM diazoxide followed by inhibition by the addition of 20 µM VU591. Lower panel: representative single-channel recordings at −70 mV under control conditions in the presence of 30 µM diazoxide and after the addition of 20 µM VU591. Lower panel: channel open probability, nP(o), in control conditions, in the presence of 30 µM diazoxide, or 30 µM diazoxide and 20 µM VU591 (*n* = 3). All modulators were applied to the *cis* and *trans* compartments simultaneously. “-” indicates the closed state of the channel. The results related to channel open probability are presented as means ± SD. * *p* < 0.05, *** *p* < 0.001.

In some additional experiments, channels activated by diazoxide added to *cis* and *trans* compartments were treated with inhibitors: VU591 and ATP/Mg^2+^. As shown in Figure 4A, channels activated with 30 μM diazoxide (an increase of the mean open probability from 0.05 ± 0.06 to 0.96 ± 0.51) were blocked by the addition (to *cis* and *trans* compartments) of 500 μM ATP/Mg^2+^ (a decrease of the mean open probability to 0.39 ± 0.40 (*n* = 8)). Similarly, Figure 4B shows that the addition of VU591 blocked the channel previously activated with 30 µM diazoxide reducing the open probability from 1.08 ± 0.56 to 0.06 ± 0.06 (*n* = 3).

**Figure 5:**
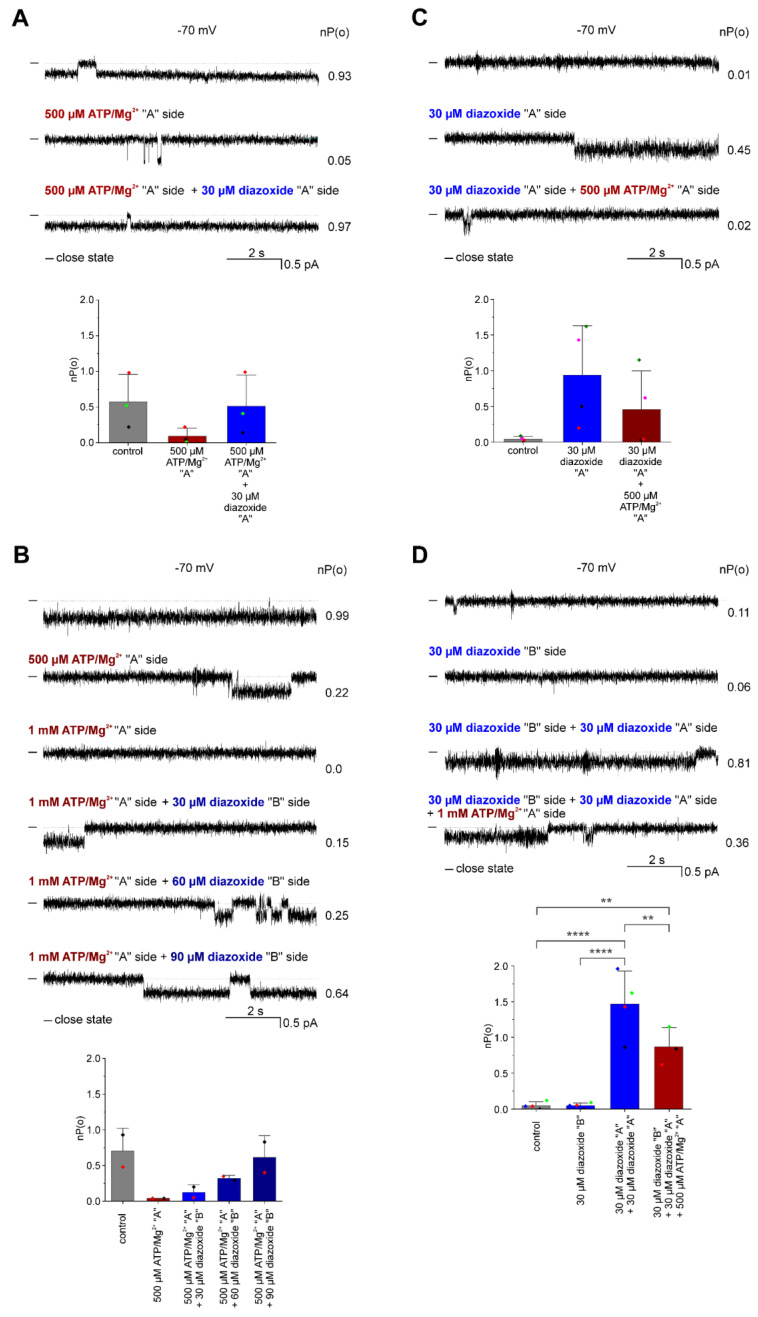
The polarity of the effect of diazoxide and ATP/Mg^2+^ on ROMK2 channel activity. (**A**) Immediate activation of ATP-inhibited ROMK2 channels by same-side addition of diazoxide. Top panel: representative single-channel recordings at −70 mV in control conditions and in the presence of 500 µM ATP/Mg^2+^ at one side (“A” side), followed by the addition of 30 µM diazoxide to the same side. The “A” side was defined as the one from which the inhibitory effect of ATP/Mg^2+^ was observed. Lower panel: channel open probability, nP(o), under control conditions, after the addition of 500 µM ATP/Mg^2+^ from the “A” side, followed by the addition of 30 µM diazoxide from the same side (*n* = 3). (**B**) Delayed effect of activation of ATP-inhibited ROMK2 channel by opposite-side addition of diazoxide. Top panel: representative single-channel recordings at −70 mV under control conditions and in the presence of 500 μM ATP/Mg^2+^ on one side (“A” side), followed by the addition of different concentrations of diazoxide (30 μM, 60 μM, and 90 μM) to the other side (“B” side). Lower panel: channel open probability, nP(o), under control conditions, in the presence of 500 μM ATP/Mg^2+^ on one side, followed by the addition of increasing concentrations of diazoxide (30 μM, 60 μM, and 90 μM) to the other side (*n* = 2). (**C**) Inhibition of diazoxide-activated ROMK2 channels by same-side addition of ATP/Mg^2+^. Top panel: representative single-channel recordings at −70 mV in control conditions and in the presence of 30 µM diazoxide on one side, followed by the addition of 500 µM ATP/Mg^2+^ to the same side. The “A” side was defined as the one from which the activatory effect of diazoxide was observed. Lower panel: channel open probability (nP(o)) under control conditions and in the presence of 30 µM diazoxide on one side (“A” side), followed by the addition of 500 µM ATP/Mg^2+^ to the same side (*n* = 4). (**D**) The polar effect of diazoxide activation correlates with the side of ATP/Mg^2+^ inhibition. Top panel: Representative single-channel recordings at −70 mV under control conditions and in the presence of 30 µM diazoxide (“B” side), followed by the addition of 30 µM diazoxide to the other side (“A” side) and the addition of 500 µM ATP/Mg^2+^ to the side of diazoxide action (“A” side). Lower panel: channel open probability, nP(o), under control conditions, in the presence of 30 μM diazoxide on one side (“B” side), followed by the addition of 30 μM diazoxide to the other side (“A” side), followed by the addition of 500 µM ATP/Mg^2+^ to the same side (*n* = 4). (experiments in symmetrical conditions. “-” indicates a closed state of the channel. The results related to channel open probability are presented as means ± SD. ** *p* < 0.01, **** *p* < 0.0001.

In a subset of experiments, we investigated the polarity of diazoxide and ATP/Mg^2+^ action on the ROMK2 channels (Figure 5). The ATP binding site in ROMK2 can be inferred from homology with other Kir channels [11,29,30]; therefore, comparing the effects of both substances could establish the side of the channel at which diazoxide is active. For this purpose, the ATP/Mg^2+^ was sequentially added to the *cis* and *trans* compartments, followed by the addition of the diazoxide. In this case, we did not use the “cis” and “trans” descriptors used for the physical sides of the PLB setup and instead designated sides as “A” and “B”. We defined the “A” side as the one from which the inhibitory effect of ATP/Mg^2+^ was observed and labeled the opposite side as the “B” side. In three of five experiments, the addition of ATP/ Mg^2+^ decreased the open probability of the channel from 0.57 ± 0.38 to 0.09 ± 0.11, and the addition of 30 µM diazoxide to the same side immediately increased the open probability of the channel to 0.51 ± 0.43 (Figure 5A). In the remaining two experiments, diazoxide activated the channel when added to the opposite side than ATP/Mg^2+^ (“B” side) but at higher concentrations and delayed effect (Figure 5B), suggesting that diffusion through the membrane of this relatively hydrophobic molecule (logP = 1.2) was required for the effect to take place. In these experiments, the addition of the 500 µM ATP/Mg^2+^ resulted in a decrease in the open probability of the channel from 0.71 ± 0.32 to 0.04 ± 0.00. The opposite-side (“B” side) addition of 30 µM diazoxide increased the open probability of the channel to 0.13 ± 0.11 and 60 µM diazoxide to 0.32 ± 0.04. Only 90 μM diazoxide caused an increase in the open probability of channel to the control level (0.62 ± 0.30) (Figure 5B). A few experiments were also executed in the reverse order, i.e., starting with the addition of diazoxide. Diazoxide was sequentially added into the *cis* and *trans* compartments, followed by the addition of the ATP/Mg^2+^. In this case, we defined the “A” side as the one from which the activatory effect of diazoxide was observed and labeled the opposite side as the “B” side. Figure 5C shows that in some experiments, the first addition of 30 µM diazoxide to one side (“A” side) activated the ROMK2 channel (the open probability of the channel increased from 0.04 ± 0.04 to 0.94 ± 0.69), and then the addition of 500 µM ATP/Mg^2+^ complex to the same side decreased the open probability to 0.46 ± 0.54 (*n* = 4). In other experiments, the first addition of 30 μM diazoxide did not affect channel activity (“B” side), but after the addition of diazoxide to the opposite side (“A” side), the open probability of the channel increased from 0.05 ± 0.05 to 1.47 ± 0.46 (*n* = 4). When 500 µM ATP/Mg^2+^ was added to the “A” side, the nP(o) decreased to 0.87 ± 0.27 (*n* = 3). Hence, these results suggest that diazoxide and ATP/Mg^2+^ exert opposite effects by binding at the same membrane side of the ROMK2 channel.

**Figure 6:**
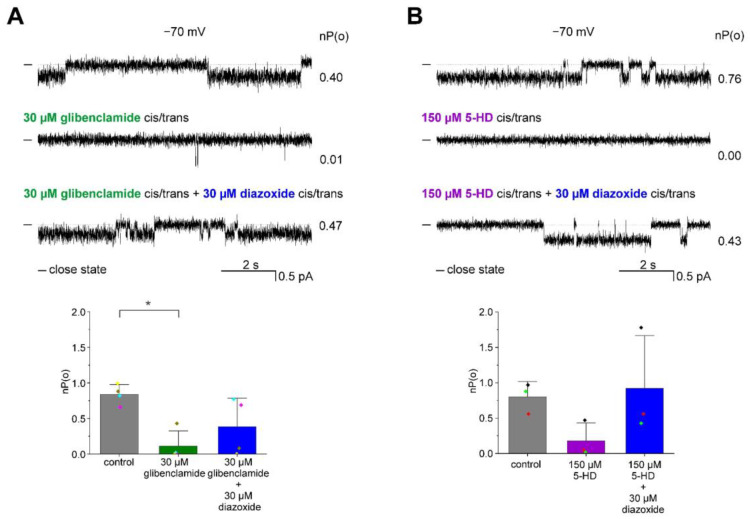
Inhibition of ROMK2 channel activity by glibenclamide and 5-HD. (**A**) Glibenclamide inhibits the activity of the ROMK2 channel. Top panel: Representative single-channel recordings at −70 mV under control conditions, in the presence of 30 µM glibenclamide followed by the addition of 30 µM diazoxide. Lower panel: Channel open probability, nP(o), under control conditions, in the presence of 30 µM glibenclamide and after the addition of 30 µM diazoxide (*n* = 4). (**B**) 5-HD blocks the activity of the ROMK2 channel. Top panel: Representative single channel recordings at −70 mV under control conditions, in the presence of 150 µM 5-HD and after the addition of 30 µM diazoxide. Lower panel: Channel open probability, nP(o), in control conditions, in the presence of 150 µM 5-HD, or 150 µM 5-HD and 30 µM diazoxide (*n* = 3). “-” indicates a closed state of the channel. The results related to channel open probability are presented as means ± SD. * *p* < 0.05.

Sulfonylurea glibenclamide has been shown to inhibit the activity of both mitoK_ATP_ channels [2] and channels formed by ROMK protein [11] in high micromolar concentrations. While previous studies have suggested that glibenclamide interacts with regulatory subunits from the SUR family to exert its effects on the ROMK channel [31], the findings of Konstas et al. [32] indicate that these subunits are not necessary for glibenclamide sensitivity. In order to investigate the direct effects of glibenclamide on ROMK channel activity, we employed the use of heterologously produced ROMK2 protein in *E. coli*. This approach eliminated the potential influence of endogenous regulatory proteins. Our results revealed that glibenclamide exhibited inhibitory effects on ROMK2 channel activity in the micromolar concentration range (Figure 6A). Specifically, the mean open probability decreased from 0.84 ± 0.14 in control conditions to 0.11 ± 0.21 in the presence of 30 μM glibenclamide (*n* = 4) (Figure 6A). This inhibition could be overcome by the subsequent addition of 30 μM diazoxide (Figure 6A).

5-Hydroxydecanoic acid (5-HD) is a well-established inhibitor of K_ATP_ channels [33], with previous studies indicating that mitoK_ATP_ may be more sensitive to this compound than the plasmalemmal K_ATP_ channels [33,34]. Additionally, it has been reported that ROMK2 channels present in mitochondrial are also sensitive to 5-HD [11,18]. To investigate the effect of 5-HD on the activity of purified ROMK2 channel protein, we conducted experiments, as shown in Figure 6B. Our results indicate that the addition of 150 μM 5-HD led to a decrease in the open probability from 0.80 ± 0.22 to 0.18 ± 0.25. Furthermore, this effect could be reversed by the addition of 30 μM diazoxide, which increased the open probability to 0.92 ± 0.74 (*n* = 3) (Figure 6B).

## 4. Discussion

Mitochondria play an important role in the energy metabolism of the cell as well as in the regulation of cell life and death. One of the mitochondrial ion channels that contributes to this regulation is the ATP-regulated mitochondrial potassium channel (mitoK_ATP_). The mitoK_ATP_ channel has been identified in mitochondria from various tissues [35]; however, it remains challenging to associate it with a specific protein. The ROMK2, which is the product of alternative splicing of the KCNJ1 gene transcript, has been identified as a potential mitoK_ATP_ channel subunit [18]. In this study, we characterized the pharmacological properties by heterologously expressing it in *E. coli* and partially purifying ROMK2. We found that the conductance of the purified ROMK2 channel protein was 11 pS ± 0.44 pS in the symmetrical conditions and 9.0 pS ± 0.92 pS in unsymmetrical conditions. In contrast, the activity of the ROMK protein recorded from the cell membranes of *Xenopus laevis* oocytes and mammalian cells was observed as a channel with a conductance of about 35 pS [36,37,38,39], whereas the conductance of the ROMK2 channel overexpressed in mitochondria of cardiac H9c2 cells was around 94 ± 3 pS [11]. These variations in the conductance of the ROMK2 channels may be due to the presence or absence of accessory proteins, posttranslational modifications, or variations in the composition of membrane lipids. Previous studies have demonstrated that the activity of Kir channels is specifically dependent on phosphatidylinositol-4,5-bisphosphate (PIP2) [40] and non-specifically on anionic phospholipids [41,42,43]. Additionally, it has been found that increasing the concentration of anionic phospholipids in the lipid bilayer affects not only the open channel probability but also increases the unitary conductance of the Kir2.1 channels [42]. Furthermore, the acyl chain length of the phospholipids could also impact channel unitary currents, as observed for KirBac3.1 [44].

Pharmacological properties are crucial for determining the type of ion channel being measured. At present, the most specific commercially available inhibitor of ROMK activity is VU591 [25,26]. The inhibition of observed single-channel activities by this modulator indicated that they are associated with the ROMK2 protein.

Another known inhibitor of the ROMK channel is ATP [11,45]. Previous studies have shown that the inhibition of plasmalemmal ROMK1 channel activity by ATP is dependent on its association with the cystic fibrosis transmembrane conductance regulator (CFTR) [31,46]. However, it has been found that the binding of CFTR to ROMK1 is dependent on N-terminal amino acids that are absent in the ROMK2 isoform. In contrast, McNicholas et al. [29] discovered that mutations in the predicted ATP-binding (Walker) motif affect the inhibition of ROMK2 by ATP, indicating a direct interaction of this nucleotide with the channel. Subsequent studies have also directly shown the binding of ATP to the C-terminal part of the protein [47]. Notably, ATP inhibition was found to be stronger for the ROMK2 isoform than for ROMK1. These studies suggest that both isoforms are inhibited by ATP, with ROMK2 being directly inhibited, while ROMK1 is inhibited in a complex with CFTR. However, it should be noted that all of the above experiments were conducted using *Xenopus laevis* oocytes leaving open the possibility of an association of ROMK2 with some endogenous proteins. Our experiments ultimately rule out this possibility and demonstrate that ROMK2 channel activity is indeed directly inhibited by micromolar ATP.

Diazoxide, a potassium channel opener, is commonly used as a vasodilator in the treatment of acute hypertension. Additionally, it can inhibit insulin secretion by opening the K_ATP_ channel of pancreatic beta cells to counter hypoglycemia in insulinoma (an insulin-producing tumor) [48]. These K_ATP_ channels are formed by Kir6.2 and regulatory sulfonylurea receptor (SUR) subunits, which bind diazoxide [49]. The cardioprotective properties of diazoxide have also been reported [28] and are thought to be associated with its binding to mitoSUR subunits of mitoK_ATP_ channels [50].

Recent research suggests that these channels may be formed by CCDC51 (mitoK) protein with SUR paralog ABCB8 (mitoSUR) [14] and that the diazoxide sensitivity of these channels is due to mitoSUR. However, diazoxide may have multiple molecular targets [51], including ATP synthase in mitochondria [20,52].

Using the patch-clamp technique, it was previously demonstrated that ROMK2 channels from cardiac H9c2 cells overexpressing ROMK2 were activated by diazoxide [11]. However, it was not determined if these effects of diazoxide were direct or related to an endogenous regulatory protein such as SUR. Our results show that diazoxide increases the open probability of purified ROMK2 channels without the involvement of any regulatory subunits and that diazoxide and ATP regulate the ROMK2 channel from the same side of the membrane. It is known that the ATP-binding site is located in the mitochondrial matrix [11], which suggests that the observed effect is related to the ATP diffusion into the interior of the mitochondria. The calculated octanol/water partition coefficient (logP) for diazoxide is 1.2 (XLogP3 3.0), which suggests high permeability across lipid membranes. The diazoxide effect on the activity of the ROMK2 channel added from the opposite side to the side on which ATP was added was also observed but at much higher concentrations and after a long time, potentially due to the permeation of diazoxide through the lipid membrane.

Pharmacological characterization of the mitoK_ATP_ channel often involves also examining the effect of the K_ATP_ channel inhibitor glibenclamide. Glibenclamide, at nanomolar concentrations, has been shown to inhibit plasmalemmal K_ATP_ channels composed of Kir6.2 and SUR1/SUR2B by binding to the SUR subunit [53]. However, at higher concentrations (in the micromolar range), glibenclamide has been observed to inhibit ROMK channel activity. It has been debated whether the presence of a SUR-type subunit is necessary for glibenclamide to regulate channel activity. Lu et al. [31] suggest that the effect of glibenclamide is dependent on the presence of CFTR protein, while Konstans et al. [32] propose that SUR-type subunits are not essential. The results presented in this study seem to confirm the latter hypothesis. Additionally, it was observed that the inhibitory effect of glibenclamide on the ROMK2 channel activity was reversible by the known mitoK_ATP_ channel activator—diazoxide.

In this study, we observed that the activity of the ROMK2 channel was blocked by 5-HD, a known inhibitor of the mitoK_ATP_ channel [54,55,56]. It confirms previous findings using the patch-clamp technique from mitoplasts isolated from cardiac H9c2 cells [11].

The study has a limitation in using single concentrations of modulators in combination, which makes quantitative interpretation impossible and provides no information about the mechanism of action. However, this approach can still provide valuable insights into the polarity of modulator action and assist in determining the molecular identity of the channel. Similar experimental methods have been previously employed in the study of mitoKATP channels, which are challenging to record [27,57,58]. Further experiments of this nature are required to address uncertainties regarding the mechanism of action of some of these modulators.

In conclusion, our results indicate that the pharmacological profile of the ROMK2 channel purified with polymer nanodiscs from bacterial cells is similar to those of the ROMK2 and mitoK_ATP_ channels previously described for mammalian cells. Our findings also support the hypothesis that the ROMK2 protein may be a component of the mitoK_ATP_ channel [18]. Furthermore, our studies have shown that the influence of modulators is direct and independent of accessory proteins of ROMK2 channels. The experimental system used in this work can be applied effectively in further studies to determine binding sites for activators and inhibitors of the ROMK2 channel.

## Data Availability

The data presented in this study are available on request from the corresponding author.
